# Clonal Dynamics and Antimicrobial Resistance of Bloodstream Carbapenem-Resistant *Acinetobacter baumannii* Isolates from Korean Hospitals Between 2016 and 2020

**DOI:** 10.3390/antibiotics15030269

**Published:** 2026-03-05

**Authors:** Young Ah Kim, Wook-Jong Jeon, Yoo Jeong Kim, Ju Hui Seo, Younggwon On, Song-mee Bae, Dong Chan Moon

**Affiliations:** 1 Department of Laboratory Medicine, National Health Insurance Service Ilsan Hospital, Goyang-si 10444, Gyeonggi-do, Republic of Korea; yakim@nhimc.or.kr; 2 Division of Antimicrobial Resistance Research, National Institute of Health, Korea Disease Control and Prevention Agency, 187 Osongsaengmyeong 2-ro, Osong-eup, Heungdeok-gu, Cheongju-si 28159, Chungcheongbuk-do, Republic of Korea; finalfate@korea.kr (W.-J.J.); yyjj0203@korea.kr (Y.J.K.); hi0327@korea.kr (J.H.S.); on0740@korea.kr (Y.O.); songmee@korea.kr (S.-m.B.)

**Keywords:** *Acinetobacter baumannii*, carbapenem-resistant, bloodstream infection, sequence type, antimicrobial resistance

## Abstract

**Background/Objective**: *Acinetobacter baumannii* is an opportunistic pathogen responsible for various healthcare-associated infections, particularly in critically ill patients. The emergence and rapid spread of multidrug- and extensively drug-resistant strains, notably carbapenem-resistant *A. baumannii* (CRAB), threaten global health. We aimed to investigate the clonal distribution, antimicrobial resistance profiles, and resistance determinants of CRAB bloodstream isolates in Korean hospitals to identify emerging high-risk clones and their potential clinical impact. **Methods**: Sequence types (STs) were determined using the Oxford multilocus sequence typing scheme, and antimicrobial susceptibility profiles and resistance determinants were evaluated. **Results**: We analyzed 812 CRAB bloodstream isolates collected from nine South Korean tertiary hospitals between 2016 and 2020. The isolates were classified into 39 STs, with ST191 (*n* = 245) and ST369 (*n* = 192) being the most prevalent. Between 2016 and 2020, ST369 increased from 2.6% to 37.9%, while ST195, first detected in 2018 (0.5%), increased to 19.0%; however, ST191 declined from 45.2% to 19.0%. Most CRAB infections were hospital-acquired (91.6%, 744 of 812), predominantly affecting men aged ≥51 years, particularly the 71–80-year-olds. Resistance rates were ≥80% for ampicillin-sulbactam and ciprofloxacin. *bla*_OXA-23_ was detected in 807 isolates, confirming its central role in carbapenem resistance. ST195 exhibited higher resistance to minocycline (29.4%) than did the other STs. **Conclusions**: Dynamic clonal shifts and high antimicrobial resistance exist among CRAB isolates in Korean hospitals, with the rapid emergence of ST195 and ST369 increasing clinical challenges. Continuous epidemiological surveillance and targeted infection control measures are essential to control the spread of high-risk CRAB clones.

## 1. Introduction

*Acinetobacter baumannii* is a clinically significant opportunistic pathogen that causes various healthcare-associated infections, including ventilator-associated pneumonia, meningitis, bloodstream infections (BSIs), urinary tract infections, and skin and soft tissue infections, particularly in critically ill patients [1,2]. Owing to the multidrug resistance (MDR) property of *A. baumannii*, carbapenems have traditionally been the most effective therapeutic option [3]. However, the rapid emergence and global dissemination of carbapenem-resistant *A. baumannii* (CRAB) pose a significant public health challenge [4]. Recognizing this threat, the World Health Organization has classified CRAB as a “critical priority pathogen” requiring the urgent development of novel antibiotics [5]. This classification is largely due to the high mortality associated with CRAB infections. In the absence of appropriate antimicrobial therapy, the 28-day mortality rate of CRAB bloodstream infections has been reported to be as high as 69.8% [6]. Although treatment options such as colistin have been attempted, they are associated with substantial toxicity. For instance, nephrotoxicity has been reported in 65.3% of patients treated with colistin compared to 39.0% in the control group. Moreover, colistin monotherapy has failed to demonstrate a significant clinical benefit [7].

CRAB-induced BSIs are associated with high morbidity and mortality, and the antimicrobial resistance (AMR) patterns of causative isolates vary geographically. The difference is influenced by local antimicrobial usage, healthcare infrastructure, and infection prevention policies. In South Korea, sequence type (ST) 191 has historically been the predominant lineage among CRAB BSI isolates [8]. A multicenter study conducted between 2016 and 2017 indicated that ST191 constituted 40% of CRAB BSI isolates and was associated with a high 30-day mortality rate [9]. Notably, in one tertiary care hospital, ST369 rapidly replaced ST191 as the dominant clone after August 2017, correlating with increased growth rates, competitive fitness, virulence, and higher early mortality [9].

Carbapenem resistance in *A. baumannii* is primarily mediated by *OXA*-type class D β-lactamases, particularly *bla*_OXA-23_, which is frequently associated with insertion sequences such as *IS*Aba1 [10,11]. In Greek hospitals, carbapenem resistance in *A. baumannii* has likewise been predominantly attributed to the production of *bla*_OXA-23_ carbapenemase [12]. Furthermore, an analysis of 842 CRAB infection cases collected from 46 hospitals across five global regions between 2017 and 2019 demonstrated that carbapenemase genes were identified in 91% (*n* = 769) of isolates, with *bla*_OXA-23_ accounting for 88% (*n* = 680) of the total cases [13]. Ambler class B metallo-β-lactamases, including *bla*_IMP_, *bla*_NDM_, and *bla*_VIM_, contribute less frequently to carbapenem resistance [11,14]. Consistent with this, Wang et al. reported that *bla*_OXA-24/40_ was detected in 9% (*n* = 75) of isolates, whereas other carbapenemase genes, including *bla*_NDM-1/OXA-58/OXA-134/OXA-237_ were identified in only 3% (*n* = 24) of cases [13]. In Korea, *bla*_OXA-23_ is detected in nearly all CRAB isolates, regardless of clonal background [15].

Analyzing clonal changes over time is important because it helps us understand the epidemiology and resistance patterns of this clinically significant pathogen. However, long-term studies on clonal shifts among carbapenem-resistant isolates from bloodstream infections are still limited, both in Korea and worldwide. Since changes in clonal distribution and resistance characteristics can directly affect clinical treatment decisions, we conducted this study to better understand these trends.

Given the clonal shifts and the clinical implications of dominant lineages, we aimed to analyze 812 CRAB BSI isolates collected from nine tertiary hospitals across South Korea between 2016 and 2020. Using the Oxford multilocus sequence typing (MLST) scheme, we sought to evaluate antimicrobial susceptibility profiles, resistance determinants, and their associations with MLST types, to characterize the epidemic potential of major CRAB clones and inform clinical management and infection control strategies.

## 2. Results

### 2.1. Epidemiological Characteristics of CRAB Isolates

Among the 812 CRAB isolates, 744 (91.6%) were classified as hospital-acquired, and 68 (8.4%) were community-acquired. All 51 ST195 isolates were of hospital origin (Table S1). Isolates were more frequently obtained from male patients (*n* = 533) than from female patients (*n* = 279), with approximately a two-fold higher isolation rate in males across most STs, except for ST191 (Table S2). By department, the highest number of patients were from pulmonology, and overall, isolates were obtained more than twice as often from ICU patients compared to non-ICU patients (Tables S3 and S4). Regarding age distribution, 88.2% (*n* = 716) of infections occurred in patients aged >50 years, with the highest prevalence observed in the 71–80-year age group (33.0%, *n* = 268). No cases of ST369 or ST451 infections were detected in patients aged <20 years (Table S5).

### 2.2. Distribution and Temporal Trends of STs

**Figure 1:**
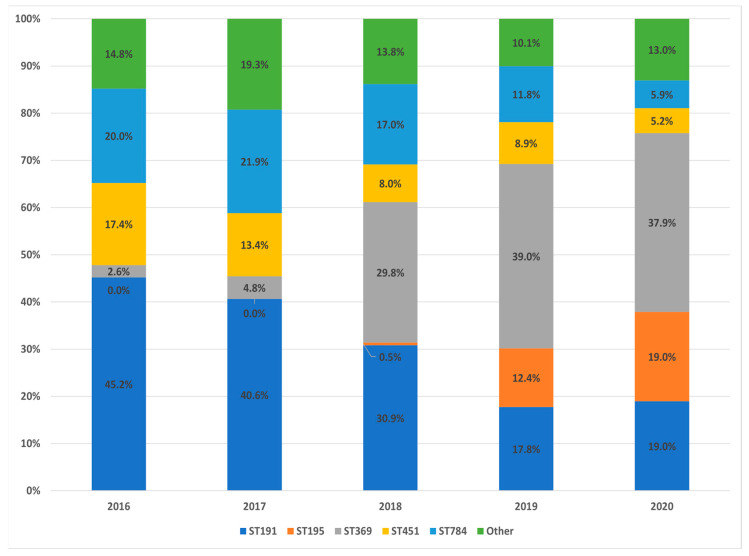
Annual distribution of carbapenem-resistant *Acinetobacter baumannii* isolates between 2016 and 2020.

The 812 CRAB isolates were assigned to 39 STs using the Oxford MLST scheme. The most prevalent STs were ST191 (*n* = 245) and ST369 (*n* = 192), both constituting 53.8% of all isolates. Other major STs included ST784 (*n* = 125), ST451 (*n* = 83), ST195 (*n* = 51), and a group of diverse STs (*n* = 116). Six isolates were identified as novel STs: ST3364, ST3365, ST3371, ST3372, ST3373, and ST3374 (Tables S1 and S6). Between 2016 and 2020, marked clonal shifts were observed. The proportion of ST369 increased from 2.6% in 2016 to 37.9% in 2020 (Figure 1). In addition, ST195, first detected in 2018 (0.5%), increased sharply to 19.0% in 2020. In contrast, ST191 declined from 45.2% in 2016 to 19.0% in 2020. ST784, the second most common ST in 2016 (20.0%), decreased to 5.9% in 2020, and ST451 declined from 17.4% to 5.2% over the same period. Other STs, which accounted for 14.8% of the isolates in 2016, fluctuated slightly, ranging between 19.3% and 10.1% in subsequent years.

### 2.3. Antimicrobial Susceptibility of A. baumannii Isolates

Among 911 *A. baumannii* isolates, 99 (10.9%) were susceptible to carbapenems. Carbapenem-resistant strains exhibited ≥80% resistance to ampicillin-sulbactam and ciprofloxacin, as well as over 76% resistance to aminoglycosides (amikacin, gentamicin, and tobramycin) (Table 1). In contrast, lower resistance rates were observed for piperacillin-tazobactam (18.8%), tigecycline (6.8%), minocycline (3.7%), and colistin (1.8%). Notably, ST195 showed markedly higher resistance to piperacillin-tazobactam (56.9%) than did ST369 (30.2%), as well as elevated resistance to minocycline (29.4%) and tigecycline (13.7%). In addition, compared with CRAB isolates of other STs, ST195 exhibited significantly higher resistance rates to five antimicrobial agents.

### 2.4. Resistance Genes of Carbapenems in A. baumannii Isolates

Among the 812 carbapenem-non-susceptible isolates, the *bla*_OXA-23_ genes were detected in 807 isolates, respectively. Five isolates lacked *bla*_OXA-23_ (Table 2).

## 3. Discussion

In the present study, 89.1% (812 of 911) of *A. baumannii* infections were caused by CRAB. Notably, 91.6% (744 of 812) of the CRAB cases were hospital-acquired, indicating the significance of nosocomial transmission in *A. baumannii* infections in Korea. Moreover, all ST195 isolates were of hospital origin, validating this observation. In addition, ST195 belongs to an ST191 subgroup and participates in nosocomial infections in China and Hong Kong [16,17,18]. CRAB infections were predominantly detected in older male patients, particularly those aged ≥51 years, with the highest prevalence in the 71–80-year age group. The finding that approximately 70% of CRAB infections occurred in men is consistent with reports from China, validating the increased susceptibility of older men to CRAB infections and the need for adequate clinical attention [19,20]. In addition, CRAB infections were most frequently observed in patients from pulmonology, internal medicine, and neurosurgery, and occurred more than twice as often in ICUs compared to general wards, indicating that CRAB is a significant pathogen particularly among critically ill patients with severe respiratory diseases.

This study is significant because it reveals clonal shifts in CRAB isolates collected from nine Korean hospitals between 2016 and 2020. Among the 39 multilocus STs identified, five major types (ST191, ST195, ST369, ST451, and ST784) predominated. While ST191, ST451, and ST784 are historically common in Korea and remain reported in other countries, their prevalence in bloodstream CRAB isolates has declined over time. In contrast, ST195 and ST369 have shown marked increases recently. Similar patterns have been observed internationally: ST191 prevalence is decreasing in Hong Kong and China, whereas ST195 and ST208 are emerging as dominant clones [18,21,22]. Although ST208 has been detected in Korean bloodstream isolates, its spread remains limited, as 12 of 17 isolates were identified in two hospitals in 2021. 

The prevalence of ST451 fluctuates, decreasing in China before reemerging in 2019 [23] and being consistently detected in Thailand [24] and India [25]. However, the prevalence of ST451 gradually declined from 17.4% in 2016 to 5.2% in 2020 in Korea. Furthermore, ST784 was an emerging clone in Korea between 2016 and 2018 [26]. Consistent with these reports, our study demonstrated that ST784 constituted 20.0–17.0% of isolates between 2016 and 2018; however, its prevalence gradually declined to 11.8% in 2019 and 5.9% in 2020.

The rapid expansion of ST195 and ST369 in Korea is noteworthy, as both clones originate from ST191 [16]. ST195 was first detected in 2018 (0.5%, 1 isolate) and increased sharply to 19.0% (29 isolates) in 2020. Belonging to international clone 2, ST195 harbors multiple virulence factors, including *OXA-23* production, biofilm formation, OmpA, PGA, phospholipase C/D, and iron acquisition genes, contributing to its high pathogenic potential [16,27].

Additionally, ST369 has expanded rapidly since 2018, replacing ST191 as the dominant ICU clone within approximately 8 months in one Korean hospital [9]. Chinese reports indicate that ST369 exhibits extensive antimicrobial resistance and high virulence, facilitating its rapid spread [28]. Experimental mouse models have shown higher lethality for ST369 than for ST191 and ST784 [9].

Over 79.1% of CRAB isolates in this study were multidrug-resistant (i.e., resistant to ≥3 antibiotic classes). In particular, ST195 showed higher resistance rates to minocycline (29.4%) than did the other STs. Tigecycline, a minocycline derivative that inhibits protein synthesis, remains a key alternative treatment option for CRAB infections [26,29]. The emergence of minocycline-resistant ST195 strains has increased the risk of selection and persistence of these high-risk clones in hospitals. Although ST369 and ST195 are increasing, it remains unclear whether this reflects expansion of a single clone or multiple distinct lineages. Therefore, enhanced epidemiological surveillance, including WGS-based analysis, is essential to better define their spread.

Consistent with previous Chinese and Korean reports [2,16], *bla*_OXA-23_ was identified as the predominant carbapenem resistance gene, present in 99.4% (807/812) of CRAB isolates. Continued surveillance and characterization of epidemic clones are essential to prevent the further dissemination of CRAB.

## 4. Materials and Methods

### 4.1. Bacterial Isolates

A total of 911 *A. baumannii* isolates were collected from nine Korean sentinel hospitals participating in Kor-GLASS (Korea Global Antimicrobial Resistance Surveillance System) between 2016 and 2020. BSI cases were analyzed between January 2016 and December 2020. The number of participating hospitals varied by year: six in 2016, eight in 2017–2019, and nine in 2020.

Species identification was performed using matrix-assisted laser desorption/ionization time-of-flight mass spectrometry (Bruker Microflex LT, Bremen, Germany) and *rpoB* sequencing [30].

### 4.2. Antimicrobial Susceptibility Testing

Antimicrobial susceptibility was determined using the disk diffusion method, according to Clinical and Laboratory Standards Institute guidelines CLSI M100 [31]. Susceptibility to colistin was determined via broth microdilution using the Sensititre KNIHCOL system (Thermo Fisher Scientific, East Grinstead, UK). Eleven antimicrobial agents were tested: imipenem, meropenem, ampicillin-sulbactam, piperacillin-tazobactam, amikacin, gentamicin, tobramycin, ciprofloxacin, minocycline, tigecycline, and colistin. Diluted bacteria were dispensed into the Sensititre plate using the Sensititre AIM (Thermo Fisher Scientific), and readings were taken with the Sensititre Vizion (Thermo Fisher Scientific). *Escherichia coli* ATCC 25922 and *Pseudomonas aeruginosa* ATCC 27853 were used as quality control strains. Tigecycline susceptibility was interpreted according to the Food and Drug Administration criteria for Enterobacteriaceae. Multidrug-resistant phenotypes were classified according to the definitions by Magiorakos et al. [32].

### 4.3. Detection of Antimicrobial Resistance Genes

Isolates were grown overnight in Luria-Bertani broth, harvested via centrifugation, washed with phosphate-buffered saline, and resuspended in sterile distilled water. DNA templates were prepared by boiling bacterial suspensions for 10 min. Polymerase chain reaction (PCR) amplification targeted carbapenem resistance genes (*bla*_OXA-23_, *bla*_OXA-51_, *bla*_OXA-24_, *bla*_OXA-58_, *bla*_IMP_, *bla*_NDM_, and *bla*_VIM_), as described in the Kor-GLASS protocol [30]. PCR was performed using the Mastercycler Pro S (Eppendorf, Hamburg, Germany).

### 4.4. Sequence Typing

MLST was performed using the Oxford scheme. Seven housekeeping genes (*gltA*, *gyrB*, *gdhB*, *recA*, *cpn60*, *gpi*, and *rpoD*) were PCR-amplified and sequenced. Allelic profiles and STs were assigned using the *A. baumannii* MLST database (http://pubmlst.org/abaumannii (accessed on 2 December 2025)).

### 4.5. Statistics

Statistical analyses were performed using IBM SPSS Statistics version 29.0 (IBM, New York, NY, USA). Antimicrobial non-susceptibility rates among different sequence types (STs) were compared using the chi-square test or Fisher’s exact test. Frequency data were analyzed using the weight cases procedure. Post hoc analysis with adjusted standardized residuals was conducted to identify specific STs with significant differences, with an absolute value > 1.96 considered statistically significant (*p* < 0.05).

## 5. Conclusions

We investigated the STs of *A. baumannii* isolates, considering their AMR profiles and resistance determinants. Historically dominant clones, such as ST191, showed a marked decline over time (45.2% to 19.0%), whereas emerging clones, including ST195 and ST369, demonstrated a substantial increase in prevalence since 2018 (0.5% to 19.0% and 2.6% to 37.9%, respectively).

The prevalence of key resistance determinants, such as *bla*_OXA-23_ (99%, *n* = 807), as well as resistance to the most commonly used antibiotics, has remained largely stable over the last decade. In addition, resistance to minocycline have increased owing to the expansion of high-risk clones, particularly ST195. Enhanced surveillance and infection control measures are warranted to prevent the dissemination of carbapenem-resistant ST195 and ST369 strains.

## Figures and Tables

**Table 1:** Analysis of antimicrobial resistance determinants, ordered by strain, multilocus sequence typing (MLST) form, and the international clonal lineage of the assessed *Acinetobacter baumannii* isolates.

Antimicrobial Agents	No. (%) of Non-Susceptible Isolates							
	ST191 ^(1)^	ST195	ST369	ST451	ST784	Others	Total	*p*-Value
	(*n* = 245)	(*n* = 51)	(*n* = 192)	(*n* = 83)	(*n* = 125)	(*n* = 116)	(*n* = 812)	
Ampicillin-sulbactam	211 (86.1 ^(2)^)	49 (96.1)	161 (83.9)	74 (89.2)	105 (84.0)	105 (90.5)	705 (86.8)	0.153
piperacillin-tazobactam	29 (11.8)	29 (56.9) *	58 (30.2) *	8 (9.6)	9 (7.2)	20 (17.2)	153 (18.8)	<0.001
Amikacin	196 (80.0)	48 (94.1) *	120 (62.5)	70 (84.3)	113 (90.4) *	70 (60.3)	617 (76.0)	<0.001
Gentamicin	205 (83.7) *	48 (94.1) *	131 (68.2)	74 (89.2) *	114 (91.2) *	70 (60.3)	642 (79.1)	<0.001
Tobramycin	201 (82.0) *	48 (94.1) *	129 (67.2)	70 (84.3) *	115 (92.0) *	60 (51.7)	623 (76.7)	<0.001
Ciprofloxacin	244 (99.6)	51 (100)	192 (100)	83 (100)	125 (100)	115 (99.1)	810 (99.8)	0.675
Minocycline	3 (1.2)	15 (29.4) *	1 (0.5)	7 (8.4) *	1 (0.8)	3 (2.6)	30 (3.7)	<0.001
Tigecycline	13 (5.3)	7 (13.7)	10 (5.2)	10 (12.0)	6 (4.8)	9 (7.8)	55 (6.8)	0.073
Colistin	2 (0.8)	1 (2.0)	6 (3.1)	2 (2.4)	0 (0)	2 (1.7)	13 (1.6)	0.285

^(1)^ ST; sequence type; ^(2)^ Resistance rate to each antimicrobial agent according to ST. * *p* < 0.05.

**Table 2:** Distribution of resistance genes and antimicrobial susceptibility test results in 812 *A. baumannii* isolates based on STs.

ST	No. of Isolates Carrying the Following Genes		No. (%) of Non-Susceptible Isolates	
	*bla*_OXA-23_	*bla*_OXA-51_	Ampicillin-Sulbactam	Piperacillin-Tazobactam
ST191 (*n* = 245)	245	245	211 (86.1)	29 (11.8)
ST195 (*n* = 51)	51	51	49 (96.1)	29 (56.9)
ST369 (*n* = 192)	189	192	161 (83.9)	58 (30.2)
ST451 (*n* = 83)	81	83	74 (89.2)	8 (9.6)
ST784 (*n* = 125)	125	125	105 (84.0)	9 (7.2)
Other (*n* = 116)	116	116	105 (90.5)	20 (17.2)
Total (*n* = 812)	807	812	705 (86.8)	153 (18.8)

## Data Availability

The original contributions presented in this study are included in the article/Supplementary Material. Further inquiries can be directed to the corresponding author.

## Supplementary Materials

The following supporting information can be downloaded at: https://www.mdpi.com/article/10.3390/antibiotics15030269/s1, Table S1: Frequency of hospital- and community-acquired bloodstream *Acinetobacter baumannii* isolates from Korean hospitals between 2016 and 2020; Table S2: Sex-based distribution of bloodstream *Acinetobacter baumannii* isolates from Korean hospitals between 2016 and 2020; Table S3: Department-based distribution of bloodstream *Acinetobacter baumannii* isolates from Korean hospitals between 2016 and 2020; Table S4: ICU admission status-based distribution of bloodstream *Acinetobacter baumannii* isolates from Korean hospitals between 2016 and 2020; Table S5: Age-based distribution of bloodstream *Acinetobacter baumannii* isolates from Korean hospitals between 2016 and 2020; Table S6: Distribution of non-predominant sequence types among bloodstream *Acinetobacter baumannii* isolates from Korean hospitals between 2016 and 2020.

## Abbreviations

The following abbreviations are used in this manuscript:

CRAB	carbapenem-resistant *Acinetobacter baumannii*
STs	sequence types
BSIs	bloodstream infections
MDR	multidrug resistance
AMR	antimicrobial resistance
MLST	multilocus sequence typing
PCR	Polymerase chain reaction
