# Supplementary material for: Clonal Dynamics and Antimicrobial Resistance of Bloodstream Carbapenem-Resistant Acinetobacter baumannii Isolates from Korean Hospitals Between 2016 and 2020
Source: Antibiotics (Basel). 2026 Mar 5;15(3):269. doi: 10.3390/antibiotics15030269 (PMC13023889; doi:10.3390/antibiotics15030269)
Supplement: Supplementary file 1 [file antibiotics-15-00269-s001.zip › antibiotics-4120115-supplementary.pdf]

Supplementary Table S1. Frequency of hospital- and community-acquired bloodstream *Acinetobacter baumannii* isolates from Korean hospitals between 2016 and 2020

Sequence types (STs)	Community-acquired	Hospital-acquired	Total
191	21	224	245
195		51	51
369	19	173	192
451	10	73	83
784	8	117	125
Other	10	106	116
Total	68	744	812

Accordingly, following the **CDC/NHSN Surveillance Definition of Healthcare-Associated Infection and Criteria for Specific Types of Infections in the Acute Care Setting** and the **Kor-GLASS manual**, HAI in this study was defined using **hospital Day 3 as the cutoff** to distinguish healthcare-associated infections from community-acquired infections.

Supplementary Table S2. Sex-based distribution of bloodstream *Acinetobacter baumannii* isolates from Korean hospitals between 2016 and 2020

STs	Male	Female	Total
191	143	102	245
195	37	14	51
369	130	62	192
451	58	25	83
784	83	42	125
Other	82	34	116
Total	533	279	812

Supplementary Table S3. Department-based distribution of bloodstream *Acinetobacter baumannii* isolates from Korean hospitals between 2016 and 2020

Department	ST191	ST195	ST369	ST451	ST784	Other	Total
Admission	0	0	0	0	1	0	1
Allergy and Clinical Immunology	0	0	2	0	0	0	2
Allergy and Environmental Medicine	3	0	0	0	0	0	3
Cardiology	6	2	10	2	9	6	35
Cardiothoracic Surgery	10	13	8	6	8	8	53
Colorectal Surgery		1		1		2	4
Emergency Medicine	14	3	17	6	7	8	55
Endocrinology	1	0	1	1	0	0	3
Gastroenterology	14	3	16	5	11	11	60
General Surgery	22	2	7	5	9	5	50
Hematology-Oncology	10	3	3	6	1	6	29
Hepato-Biliary-Pancreatic Surgery	0	0	0	0	1	0	1
Infectious Diseases	19	3	9	8	11	10	60
Internal Medicine	32	6	20	5	0	4	67
Nephrology	24	2	15	4	10	8	63
Neurology	4	3	6	3	4	0	20
Neurosurgery	18	1	24	2	10	12	67
Nuclear Medicine						1	1
Obstetrics and Gynecology	0	0	0	1	0	1	2
Orthopedic Surgery	1	0	5	1	0	0	7
Otolaryngology			1				1
Pediatrics	12	1	2	3	8	3	29
Plastic Surgery						1	1
Pulmonology	41	7	44	19	26	26	163
Rehabilitation Medicine	0	0	0	0	4	1	5
Rheumatology	10	0	0	2	0	0	12
Transplantation Surgery			1				1
Trauma & Critical Care	4	1	0	3	5	2	15
Urology	0	0	1	0	0	1	2
Total	245	51	192	83	125	116	812

Supplementary Table S4. ICU admission status-based distribution of bloodstream *Acinetobacter baumannii* isolates from Korean hospitals between 2016 and 2020

STs	General wards	ICU	Unknown	Total
191	66	177	2	245
195	11	40		51
369	56	136		192
451	33	50		83
784	28	96	1	125
Other	42	73	1	116
Total	236	572	4	812

Supplementary Table S5. Age-based distribution of bloodstream *Acinetobacter baumannii* isolates from Korean hospitals between 2016 and 2020

	Age (years)										
STs	0–10	11–20	21–30	31–40	41–50	51–60	61–70	71–80	81–90	91–98	Total
191	1		4	3	19	39	59	78	41	1	245
195		1	2	3	4	3	13	16	9		51
369			4	9	7	18	37	73	37	7	192
451				2	3	17	19	26	13	3	83
784	2	2	7	4	8	16	21	37	27	1	125
Other		2			9	10	29	38	26	2	116
Total	3	5	17	21	50	103	178	268	153	14	812
%	0.4	0.6	2.1	2.6	6.2	12.7	21.9	33.0	18.8	1.7	100.0

Supplementary Table S6. Distribution of non-predominant STs among bloodstream *Acinetobacter baumannii* isolates from Korean hospitals between 2016 and 2020

Years	STs of bloodstream *Acinetobacter baumannii* isolates																																			TOTAL
	2	94	208	357	358	368	447	465	469	473	491	502	804	862	1224	1246	1492	1571	1573	1574	1599	1600	1601	1602	1890	1933	2879	2929	3364	3365	3371	3372	3373	3374	ND	
2016			5	3	1		5			1	1						1																			17
2017		2	3	2		3	8	1	1		1	1	1			1		1	1	2	1	3	1	3												36
2018			9	3			6		4						2																				2	26
2019	2			3					4		4										1							1	1	1						17
2020				3					2		4			1	1										1	1	1	1			1	1	1	1	1	20
Total	2	2	17	14	1	3	19	1	11	1	10	1	1	1	3	1	1	1	1	2	2	3	1	3	1	1	1	2	1	1	1	1	1	1	3	116
